# Multiple roles played by the mitochondrial citrate carrier in cellular metabolism and physiology

**DOI:** 10.1007/s00018-022-04466-0

**Published:** 2022-07-17

**Authors:** Vincenzo Zara, Graziana Assalve, Alessandra Ferramosca

**Affiliations:** Department of Biological and Environmental Sciences and Technologies, University of Salento, 73100 Lecce, Italy

**Keywords:** Citrate, Intermediary metabolism, Metabolite carrier, Mitochondria, Metabolic network, Subcellular compartments

## Abstract

The citrate carrier (CIC) is an integral protein of the inner mitochondrial membrane which catalyzes the efflux of mitochondrial citrate (or other tricarboxylates) in exchange with a cytosolic anion represented by a tricarboxylate or a dicarboxylate or phosphoenolpyruvate. In this way, the CIC provides the cytosol with citrate which is involved in many metabolic reactions. Several studies have been carried out over the years on the structure, function and regulation of this metabolite carrier protein both in mammals and in many other organisms. A lot of data on the characteristics of this protein have therefore accumulated over time thereby leading to a complex framework of metabolic and physiological implications connected to the CIC function. In this review, we critically analyze these data starting from the multiple roles played by the mitochondrial CIC in many cellular processes and then examining the regulation of its activity in different nutritional and hormonal states. Finally, the metabolic significance of the citrate flux, mediated by the CIC, across distinct subcellular compartments is also discussed.

## Introduction

Mitochondrial carrier proteins play an essential role in the control of the flow of metabolites between the cytosol and mitochondrial matrix, thus contributing to many cellular processes. Most of these metabolite carriers, encoded by the nuclear genes of the SLC25 family [1], have been characterized both structurally and functionally in several species [2–6]. By transporting several substrates, these proteins are involved in a variety of biochemical processes, such as Krebs cycle, oxidative phosphorylation (OXPHOS), the transfer of reducing equivalents, gluconeogenesis, fatty acid metabolism, amino acid synthesis, and cofactor transport. Therefore, the transport activities of these proteins are carefully orchestrated to channel several molecules belonging to the same metabolic pathway in a fine and coordinated manner [1, 2, 5].

In this complex metabolic network, the citrate carrier (CIC) mediates the efflux of citrate and other metabolites from the mitochondrial matrix to the cytosol. By doing so, the CIC links intra-mitochondrial and extra-mitochondrial (cytosolic) reactions in which the substrates transported by this carrier protein are involved.

The mitochondrial CIC (SLC25A1) was discovered in 1967 by Chappell and Haarhoff in rat liver mitochondria [7]. Then, the protein was purified from rat and bovine liver [8–10], yeast [11], eel [12] and maize mitochondria [13] and functionally reconstituted into liposomes to study its transport properties. The human CIC is encoded in the nucleus by the SLC25A1 gene located on chromosome 22q11.2. The protein is synthesized in the cytosol as a precursor protein with an amino-terminal presequence of 13 amino acids [14] whose role has been extensively investigated [15, 16]. The mature protein is made up of three homologous sequence domains, each of them containing ~ 100 amino acids and organized in the form of 2 α-helices. It is firmly inserted in the inner mitochondrial membrane where it catalyzes with high affinity the flux of citrate and other substrates such as isocitrate [8–10] across the lipid bilayer in strict cooperation with many catabolic and anabolic reactions occurring inside and outside mitochondria. The CIC is therefore able to modulate the amount of citrate both in the mitochondrial matrix and in the cytosolic space. This last pool of citrate also depends on the activity of a plasma membrane citrate transporter [17] which catalyzes the entry of external citrate into the cell. However, recent studies suggest that exogenous citrate appreciably contributes to intermediary metabolism only in oxygen- and glutamine-limited conditions [18].

Cytoplasmic citrate serves not only as a carbon source for fatty acid and sterol biosynthetic pathways [19], but also as a key regulator of several enzymatic activities. Moreover, a role for citrate in inflammation, insulin secretion, and histone acetylation has been proposed [20]. In this way, the mitochondrial CIC and citrate play a pivotal role in cell metabolism. Indeed, in mitochondria, citrate mainly represents an intermediate of the Krebs cycle, whereas in the cytosol, it may represent a metabolic substrate for lipogenesis [19], an allosteric enzymatic regulator, a signaling molecule and an epigenetic modifier [20].

In plants, a carrier related to CIC is the dicarboxylate-tricarboxylate carrier (DTC) that catalyzes an electroneutral exchange of protonated tricarboxylates for unprotonated dicarboxylates [4, 21]. As DTC transports a broad spectrum of dicarboxylates (oxoglutarate, oxaloacetate, malate, maleate, succinate, and malonate) and tricarboxylates (citrate, isocitrate, and aconitate), it may play a role in several important plant metabolic processes, i.e., production of glycerate, nitrogen assimilation and ripening of fruits [4].

In this review, we critically analyze the multiple roles played by the mitochondrial CIC in many cellular processes in order to highlight the complex framework of metabolic and physiological implications of this carrier protein in mammalian cells. The regulation of its activity in different nutritional and hormonal states will also be examined. We believe that the understanding of the role played by the CIC in cellular metabolism and physiology can provide useful insight into the molecular basis of the human pathologies in which this carrier protein is involved.

## Multiple roles of the mitochondrial CIC in cellular metabolism

**Fig. 1:**
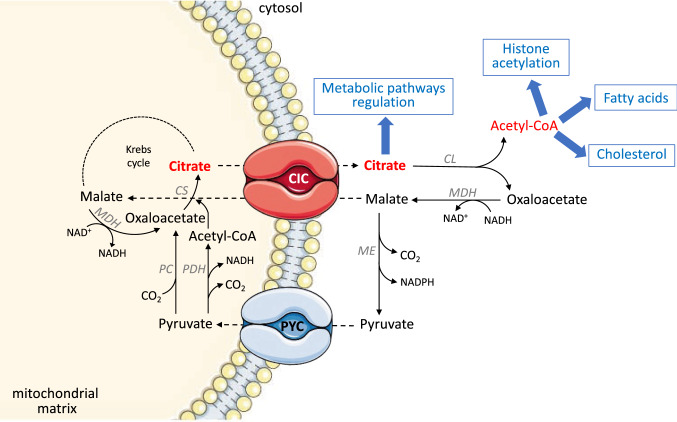
Mitochondrial CIC function. In the mitochondrial matrix, citrate synthase (CS) catalyzes the condensation of acetyl-CoA and oxaloacetate thereby generating the molecule of citrate. Citrate is transported outside mitochondria thanks to the CIC and it is broken down into acetyl-CoA and oxaloacetate, by the action of the cytosolic enzyme ATP-citrate lyase (CL). Acetyl-CoA is then channeled to the lipogenesis (fatty acids and cholesterol biosynthesis), but it can be also used to the supply of acetyl units for histone acetylation. Oxaloacetate can be reduced to malate which is redirected to mitochondria in exchange with citrate. In this way, the cytosolic malate dehydrogenase (MDH) regenerates the cytosolic NAD^+^ which is necessary for the continuous operation of glycolysis. The molecule of citrate is also a potent modulator of the activity of several enzymes involved in cellular metabolism. Malate can be transported into the mitochondrial matrix, generating NADH; alternatively, it becomes the substrate of the malic enzyme (ME) thereby generating pyruvate and NADPH. Pyruvate re-enters mitochondria by the pyruvate carrier (PYC). The mitochondrial enzyme pyruvate carboxylase (PC) converts the molecule of pyruvate into oxaloacetate, thereby stimulating the functioning of the Krebs cycle. Pyruvate dehydrogenase (PDH) can also convert pyruvate into acetyl-CoA

In the mitochondrial matrix, citrate synthase catalyzes the condensation of acetyl-CoA and oxaloacetate thereby generating the molecule of citrate which represents an intermediate of the Krebs cycle. At the high energy status in cell, i.e., high concentrations of ATP and NADH, Krebs cycle is inhibited, and citric acid is not further catabolized, but is transported outside mitochondria through CIC. Indeed, the CIC catalyzes a counter exchange of mitochondrial citrate and other negatively charged cytosolic molecules across the inner mitochondrial membrane. Outside mitochondria, ATP-citrate lyase cleaves citrate into two smaller molecules, acetyl-CoA and oxaloacetate. Acetyl-CoA is then directed toward the synthesis of fatty acids, on one hand, and/or to the cholesterol biosynthesis, on the other hand. The remaining molecule of oxaloacetate can be reduced to malate thanks to the cytosolic malate dehydrogenase and then redirected to mitochondria in exchange with citrate as depicted in Fig. 1. In this way, the malate dehydrogenase regenerates the cytosolic NAD^+^ which is necessary for the continuous operation of glycolysis. Cytosolic malate is then transported into the mitochondrial matrix, where malate oxidation generates NADH, which donates its electrons to the respiratory chain, thus stimulating OXPHOS.

Alternatively, the cytosolic malate becomes the substrate of the malic enzyme thereby generating pyruvate, which re-enters mitochondria by the pyruvate carrier (pyruvate/citrate shuttle), and NADPH which, instead, is required outside mitochondria as a source of reducing equivalents for cholesterol and fatty acid biosynthesis [22].

Interestingly, the molecule of citrate is not only the precursor of acetyl units which are important for lipid biosynthesis but is also a potent modulator of the activity of several enzymes involved in cellular metabolism (Table 1). Consequently, the level of this molecule is finely regulated [23, 24].

**Table 1:** Cytosolic and mitochondrial enzymes modulated by citrate

Metabolic pathway	Modulated enzyme	Citrate inhibition/stimulation	References
Glycolysis	PFK1	**↓**	Newsholme et al. [25]
Glycolysis	PK	**↓**	Iacobazzi and Infantino [20]
Gluconeogenesis	FBPasi1	**↑**	Taketa et al. [26]
Bifunctional enzymes	PFK2	**↓**	Chesney [27]
Lipogenesis	ACC	**↑**	Vagelos et al. [28], Kleinschmidt et al. [29]
Pyruvate decarboxylation	PDH	**↓**	Taylor and Halperin [30]
Krebs cycle	CS	**↓**	Dashty [31]
Krebs cycle	SDH	**↓**	Hillar et al. [32]

*ACC* acetyl-CoA carboxylase, *FBPasi1* fructose 1,6-bisphosphatase, *PDH* pyruvate dehydrogenase complex, *PFK1* phosphofructokinase-1, *PFK2* phosphofructokinase-2, *PK* pyruvate kinase, *SDH* succinate dehydrogenase, *CS* citrate synthase

Phosphofructokinase-1 (PFK1), the rate-limiting enzyme of glycolysis, is inhibited by high levels of citrate thus leading to a decrease in the amount of the product of this enzymatic reaction, i.e., fructose 1,6-bisphosphate [25]. Pyruvate kinase (PK), a further glycolytic enzyme, is indirectly inhibited by high levels of citrate because of the low amount of fructose 1,6-bisphosphate which is an allosteric activator of this enzyme [20]. On the contrary, fructose 1,6-bisphosphatase (FBPasi1), a key enzyme of gluconeogenesis, is stimulated by high levels of cytosolic citrate [26]. In the cytosol, there is the so-called bifunctional enzyme which contains two independent enzymatic activities in the same polypeptide chain, phosphofructokinase-2 (PFK-2) and fructose 2,6-bisphosphatase (FBPasi2). High levels of citrate inhibit the PFK-2 domain of the bifunctional enzyme thereby leading to a strong decrease in the amount of fructose 2,6-bisphosphate, an allosteric activator of PFK1 and an allosteric inhibitor of FBPase1. In this way, high levels of citrate inhibit glycolysis and stimulate gluconeogenesis [27]. Furthermore, cytosolic citrate stimulates the first step of fatty acid synthesis by promoting the formation of the active form of acetyl-CoA carboxylase (ACC) which catalyzes the synthesis of malonyl-CoA starting from the acetyl units present in the cytosol [28, 29].

Very interestingly, the molecule of citrate is also able to modulate the activity of some enzymes localized in the mitochondrial matrix. As shown in Table 1, high levels of citrate inhibit the activities of the pyruvate dehydrogenase complex (PDH) [30], of citrate synthase (CS) [31] and of succinate dehydrogenase (SDH) [32]. It is therefore evident that the molecule of citrate has an extraordinary importance in cellular metabolism because it plays a dual role as a source of carbon units for anabolic pathways and as a modulator of several biochemical reactions. In this respect, we can therefore summarize that citrate levels lead to a coordinated modulation of the metabolic pathways involved in the production and in the consumption of energy. Indeed, high levels of citrate decrease the production of ATP and NADH by inhibiting glycolysis and the Krebs cycle. On the other hand, high levels of citrate stimulate the anabolic pathways of gluconeogenesis and lipogenesis which require both ATP and NADPH to occur.

## Further roles of the mitochondrial CIC in cellular physiology

Another important role played by the mitochondrial CIC is connected to the supply of acetyl units for histone acetylation [1, 20]. Indeed, the molecule of citrate, transported outside mitochondria by the CIC, is cleaved by the cytosolic ATP-citrate lyase thus leading to the formation of acetyl-CoA which is necessary for acetylation of histone proteins [33] (Fig. 1). To achieve this effect, there must be a close cooperation between the mitochondrial CIC and the cytosolic ATP-citrate lyase.

The remodeling of nuclear chromatin, consequent to histone modification by acetylation and de-acetylation, is a crucial phenomenon linked to gene expression and DNA stability [24, 34–36]. Indeed, the silencing of CIC expression caused genome instability both in *Drosophila melanogaster* and in human fibroblasts [37]. This means that a reduced histone acetylation, due to a reduction in the citrate levels in the cytosol following to mitochondrial CIC dysfunctions, leads to the appearance of chromosomal breaks. Interestingly, histone acetylation levels are also associated with CIC expression levels [34]. In fact, experimental evidence highlights not only the key role of the acetyl-CoA-mediated chromatin remodeling in the regulation of stem cell fate decisions, but also the importance of the CIC in the metabolism-dependent chromatin rearrangements that are required for efficient cell differentiations [34]. Therefore, the transport activity of the mitochondrial CIC seems to be fundamental for genome stability [38].

Pancreatic β cells are responsible for insulin synthesis and secretion under the signal of elevated glucose concentrations in the blood. This phenomenon, known as “Glucose Stimulated Insulin Secretion” or GSIS, depends on the elevated ATP/ADP ratio induced by the complete oxidative catabolism of glucose carried out by glycolysis and then by Krebs cycle and OXPHOS. The final events are the ATP-dependent inhibition of potassium channels, the increase in cytosolic calcium followed by massive insulin secretion by pancreatic β cells [39]. In this context, an active role of pancreatic β cell mitochondria has been proposed [40]. Indeed, these organelles not only provide ATP, but also allow the synthesis of metabolites that are exported to the cytosol to support insulin secretion. A key role in this respect is, indeed, played by the mitochondrial pyruvate carboxylase (PC) which converts the molecule of pyruvate into oxaloacetate, thereby stimulating the functioning of the Krebs cycle and the production of important intermediates capable of stimulating insulin secretion. In agreement with these findings, the inhibition of PC expression lead to GSIS decrease, whereas the overexpression of PC strongly stimulated GSIS by β cells [41]. A suggestive role in GSIS is also played by the mitochondrial CIC with at least two mechanisms described below.

**Fig. 2:**
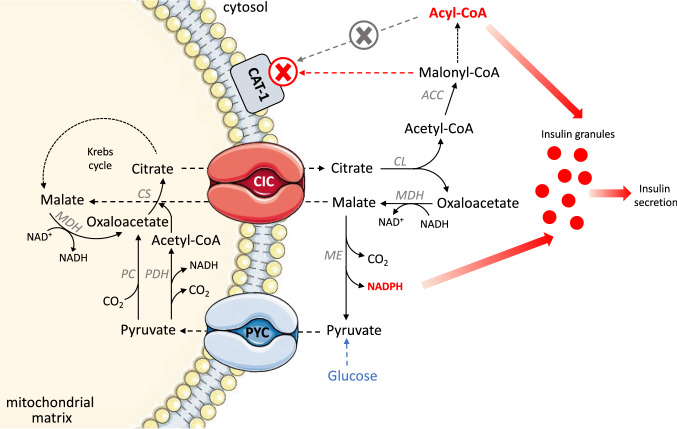
Pyruvate/citrate shuttle in pancreatic β cells. High levels of oxaloacetate and acetyl-CoA in the mitochondrial matrix lead to their condensation into the molecules of citrate, which is then transported into the cytosol. The cytosolic citrate is then broken down into two distinct molecules, acetyl-CoA, and oxaloacetate. Acetyl-CoA is directed toward the carboxylation thanks to acetyl-CoA carboxylase (ACC) and the formation of malonyl-CoA, a metabolic intermediate for fatty acid synthesis. High levels of long chain acyl-CoA stimulate insulin secretion. Oxaloacetate is instead reduced to L-malate or, alternatively, becomes the substrate of the malic enzyme thereby generating pyruvate and NADPH, which is a stimulator of insulin secretion. Pyruvate re-enters mitochondria by the pyruvate carrier (PYC). The mitochondrial enzyme pyruvate carboxylase (PC) converts the molecule of pyruvate into oxaloacetate, thereby stimulating the functioning of the Krebs cycle. Pyruvate dehydrogenase (PDH) can also convert pyruvate into acetyl-CoA

The first one is linked to the operation of the pyruvate/citrate shuttle [42, 43] which involves some mitochondrial transporters and several enzymes inside and outside mitochondria (Fig. 2). The high levels of oxaloacetate and acetyl-CoA in the mitochondrial matrix lead to their condensation in the form of molecules of citrate which are then transported into the cytosol by the mitochondrial CIC. The cytosolic citrate is then broken down by ATP-citrate lyase in two distinct molecules, acetyl-CoA which is directed toward the carboxylation and the formation of malonyl-CoA, and oxaloacetate which is instead reduced to L-malate. Malonyl-CoA can therefore act as an inhibitor of the carnitine acyl-transferase 1 (CAT1) thus inhibiting the transport of cytosolic acyl-CoA molecules into the mitochondrial matrix for β-oxidation. In this way, the high levels of long chain acyl-CoA can further stimulate insulin secretion by pancreatic β cells (Fig. 2). On the other hand, cytosolic malate is decarboxylated by the malic enzyme, with the production of pyruvate and NADPH which, in its turn, acts as a stimulator of GSIS by β cells. The molecule of pyruvate can then re-enter mitochondria by the pyruvate carrier which, therefore, acts in co-operation with the mitochondrial CIC (pyruvate/citrate shuttle) in promoting the pancreatic insulin secretion [42–44].

**Fig. 3:**
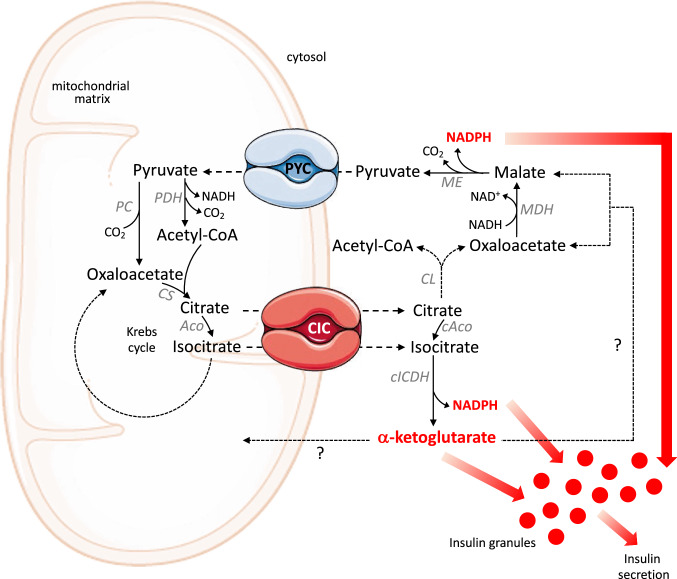
Pyruvate/isocitrate shuttle in pancreatic β cells. Citrate and isocitrate leave the mitochondrial matrix by the CIC. Citrate can be converted to isocitrate by cytosolic aconitase (cAco). The subsequent activation of the cytosolic isoform of the isocitrate dehydrogenase (cICDH) produces α-ketoglutarate and NADPH. α-ketoglutarate is a signal for insulin secretion or can be channeled into one of several mitochondrial or cytosolic pathways that remain to be defined. The other product of the pyruvate/isocitrate shuttle is the cytosolic NADPH, which is another signal for the pancreatic insulin secretion

The second mechanism is connected to the operation of the pyruvate/isocitrate shuttle [45, 46] which is reported in Fig. 3. In this second case, the efflux of citrate or isocitrate from mitochondria, mediated by the mitochondrial CIC, and the subsequent activation of the cytosolic isoform of the isocitrate dehydrogenase (cICDH) produces α-ketoglutarate and NADPH which, all together, are able to stimulate the pancreatic GSIS. According to this model, an inhibition of the transport activity of the mitochondrial CIC reduces the levels of cytosolic citrate and consequently leads to the inhibition of GSIS [46]. On the other hand, the CIC overexpression increases the levels of cytosolic citrate, which stimulate insulin secretion [47]. In summary, all these results testify the important role played by the efflux of citrate and/or isocitrate into the cytosol for an efficient secretion of insulin by the pancreatic β cells [48, 49].

It has been widely demonstrated the fundamental role played by mitochondria in the energetic metabolism of spermatozoa both in basic functions [50–52] and during capacitation [53, 54]. Spermatozoa obtain energy not only from glucose or fructose [55, 56], but also from citrate or lactate in conditions in which the availability of sugars is reduced [57, 58]. In particular, it has been proposed that the transport activity of the CIC is important for the initial steps of capacitation [59]. Sperm capacitation is a physiological event that allows sperm to bind and fuse with the oocyte and is characterized by a series of biochemical changes, such as phosphorylation of protein tyrosine residues and modification in plasma membrane fluidity. In this context, the sperm CIC is involved in the transport of acetyl units for biosynthesis of cholesterol, which is a modulator of sperm membrane fluidity, but also in the generation of NADPH molecules [53]. On the other hand, NADPH molecules are essential for cholesterol biosynthesis as well as for the activity of an NADPH-oxidase, which produces radical species promoting tyrosine phosphorylation [59, 60]. Another intriguing aspect is related to the sperm CIC regulation of insulin secretion, since it was demonstrated that insulin is an endogenous factor involved in the autocrine induction of the capacitation process [59]. In sperm cells, citrate has similar effects to that of glucose in inducing insulin secretion, which in turn regulates the recruitment of energetic substrates in dependence of sperm energy needs. Based on this evidence, we can conclude that the CIC strongly contributes to the acquisition of sperm fertilizing ability. Indeed, it has been demonstrated that the inhibition of the CIC in sperm mitochondria is able to reduce cholesterol efflux and protein tyrosine phosphorylation that are important for this process [59].

Moreover, Figs. 1, 2, 3 highlight that the CIC is involved in the balance of cellular NAD(H) and NADP(H). In eukaryotic cells, there are two main pyridine dinucleotide pools in the cytosol and in the mitochondria, which are essential not only in energy metabolism and in redox homeostasis, but also in cell differentiation. In fact, NAD(H) and NADP(H) levels reflect the integrated activities of anabolic and catabolic reactions, which are closely associated with organism development. In this context, resting cells exhibit high NAD^+^/NADH and low NADP^+^/NADPH redox ratios; on the opposite, in proliferating cells, the ratio of these redox species is reversed to support biomass synthesis and cell growth [61]. Essential to metabolic transformations related to cell differentiation is the central role of the molecule of citrate [62], which can be utilized via its entry into the Krebs cycle for ATP production or can be transported to the cytosol for de novo lipid biosynthesis. Although so far there is no direct evidence linking the CIC to the development regulation of organisms, we can speculate that this carrier protein influences NAD(H) and NADP(H) dynamics during developmental stages.

## Modulation of the mitochondrial CIC activity

The mitochondrial CIC represents an important point of connection between catabolic and anabolic reactions occurring in distinct subcellular compartments, such as mitochondria and cytosol. By virtue of this strategic role played inside the cells, the activity of the mitochondrial CIC is finely regulated by various hormonal (thyroid hormones and insulin) and nutritional factors (fatty acids and starvation) [63, 64]. These factors, by acting on CIC expression and/or transport activity, are able to influence the amount of citrate or other tricarboxylates transported across the inner mitochondrial membrane. Table 2 summarizes the main findings regarding the influence of nutritional and hormonal factors on the activity of the mitochondrial CIC.

**Table 2:** Hormonal and nutritional regulation of CIC

Condition	Effect on CIC transport activity	References
Diet enriched in PUFA	**↓**	Ferramosca and Zara [65]
Diet enriched in conjugated linoleic acids (CLA)	**↑**	Ferramosca et al. [73]
Starvation	**↓**	Zara and Gnoni [79]
Type 1 diabetes	**↓**	Kaplan et al. [81, 82]
Type 2 diabetes	-	Kaplan et al. [83]
Hyperthyroidism	**↑**	Paradies and Ruggiero [85]
Hypothyroidism	**↓**	Gnoni et al. [63]

A diet enriched in PUFA strongly inhibits the transport activity of the mitochondrial CIC, as well as the activity of the lipogenic enzymes, acetyl-CoA carboxylase (ACC) and fatty acid synthase (FAS) [64, 65]. This agrees with independent studies showing a significant inhibition of hepatic lipogenesis by a diet enrichment with *n*-3 and *n*-6 PUFA [66, 67]. On the other hand, it has also been demonstrated that the extent of hepatic lipogenesis inhibition is dependent on the amount of PUFA present in the diet and on the length of dietary treatment [68, 69]. The covariance of the activities of the mitochondrial CIC and of the cytosolic lipogenic enzymes found in hepatic cells can therefore be ascribed to at least two reasons. The first one is consistent with the fact that the mitochondrial CIC supplies the cytosolic space with the acetyl units necessary for fatty acid synthesis and the second one with the fact that the molecule of citrate exported from mitochondria is a positive modulator of the cytosolic ACC [65, 70]. Interestingly, in the same studies, it was found that the inhibition of the CIC activity was due to a decreased amount of the transport protein in the inner mitochondrial membrane and not to a modification of the Km of the CIC [64, 70]. Furthermore, it was found that the modulation of the CIC activity by dietary PUFA is a long-term effect, mediated by a complex series of transcriptional and post-transcriptional events [64, 71]. The structural and functional analysis of the CIC promoter revealed the presence of a PUFA responsive region (PUFA-RR) capable of binding several transcription factors such as SREBP-1c (sterol regulatory element binding protein), NF-Y (nuclear transcription factor-Y) and Sp1 (stimulatory protein 1). In particular, it was found that the main modulator of the CIC expression is SREBP-1c which exhibits a reduced transcriptional activity in animals fed with a diet enriched in *n*-3 and *n*-6 PUFA [63, 72]. The CIC transcriptional suppression is mediated by SRE (sterol regulatory element)/SREBP-1 regulatory system since *CIC* gene promoter contains an active SRE site. The role of SRE in the regulation of *CIC* gene transcription is also supported by the evidence that the overexpression of SREBP-1 is able to induce an increase in the levels of CIC transcript and protein [72].

Interestingly, not all PUFA are able to inhibit the transport activity of the mitochondrial CIC. In fact, a diet enriched in conjugated linoleic acids (CLA) significantly increased the activity of the mitochondrial CIC as well as that of the hepatic lipogenic enzymes [73]. CLA are a class of positional and geometric isomers of linoleic acid that exist naturally in food derived from ruminant animals and in dairy products mainly in the form of cis-9,trans-11-octadecadienoic acid [74]. The increased activity of the mitochondrial CIC found in these experimental conditions was ascribed to an increased expression of the transport protein due to higher levels of CIC mRNA [73]. The dietary supplementation with CLA has some beneficial effects linked to a prevention of fat accumulation in adipose tissue [75, 76] but this effect was negatively counterbalanced by an increased hepatic lipogenesis. Very interestingly, the dietary combination of CLA and pine nut oil, another dietary fat enriched in pinolenic acid (all-cis-5,9,12-octadecatrienoic acid), strongly prevented the CLA-induced fatty liver in mice [77]. Pinolenic acid is a quite unusual PUFA found in Korean and maritime pine seed oils, characterized by polymethylene-interrupted double bonds [78]. During this dietary treatment, it is further evident the close correlation between the changes in the transport activity of the mitochondrial CIC and those in the metabolic pathways to which this carrier protein supplies substrates. In particular, a peculiar two-phase behavior of the CIC transport activity was found in mice: at week 8 of dietary treatment, although the mitochondrial CIC activity and expression started to increase in CLA-fed mice, that of CLAs plus pine nut oil-fed mice started to decrease. Moreover, the changes in the activities of the lipogenic enzymes over time were consistent with those observed for the mitochondrial CIC [77].

A strong decrease in the transport activity of the mitochondrial CIC was found in starved rats in comparison with control animals [79]. Also in this case, a significant decrease in the Vmax was identified but no change in the Km value was found. A reduced export of citrate from the mitochondrial matrix to the cytosol was connected to a parallel decrease in the activity of hepatic lipogenic enzymes [79]. This was due, at least in part, to a lack of activation of cytosolic ACC by citrate. Interestingly, an opposite modulation of the CIC and lipogenic enzyme activities was observed in silver eels during starvation [80]. A possible explanation of the observed higher activity of CIC found in these experimental conditions could be, at least in part, linked to the different composition of the lipid domain surrounding the carrier protein. Indeed, the consequence of the hormonal alterations accompanying gonads maturation in the metamorphosis from yellow to silver stage is a change in the amount of cardiolipin which is necessary for the CIC transport activity [80].

In type 1 diabetic rats, characterized by the absence of insulin, a parallel decrease in the transport activity of the mitochondrial CIC and of its mRNA was clearly found in liver mitochondria [81, 82]. On the contrary, in type 2 diabetic rats, the transport activity of the mitochondrial CIC remained unaltered [83]. Interestingly, in rats in which the endogenous insulin was lacking, the exogenous administration of insulin restored the activity of the mitochondrial CIC [82]. It is therefore evident that the physiological transport activity of the mitochondrial CIC requires the presence of physiological levels of insulin. It was also found that the absence of insulin negatively affects CIC expression at a transcriptional and post-transcriptional level, and this is due to a reduced expression of SREPB-1 [20, 72]. Another important transcription factor is FOXA1 (Forkhead Box Transcription Factor) which behaves as an enhancer of CIC expression in the liver and pancreatic cells. FOXA1 in the pancreatic β cells plays a role in the transcriptional regulation of CIC and in insulin secretion [84]. Insulin increases lipid synthesis in liver by activating SREBP-1, which transcriptionally activates genes involved in fatty acid synthesis, including the *CIC* gene [63].

It was also found that the activity of the mitochondrial CIC was significantly regulated by thyroid hormones. Experiments carried out on hepatic mitochondria from hyperthyroid rats showed that the CIC transport activity was significantly stimulated [85]. The proposed mechanism responsible for the increase in the CIC activity was an alteration of the lipid microenvironment of the inner mitochondrial membrane, in which the CIC operates, as also observed in starved silver eels. Further studies demonstrated that the T3 thyroid hormone up-regulates the SREBP-1 transcription factor during hyperthyroidism and in Hep-2 cells incubated with high levels of T3 [86, 87]. It was therefore concluded that T3 stimulates hepatic lipogenesis by an increase in the SREPB-1 levels, which stimulate the transcription of the CIC and of the lipogenic enzymes [72, 88, 89]. Differently from the results obtained in hyperthyroid animals, a decrease in the CIC transport activity was observed in the liver of hypothyroid rats [63]. These studies indicated that the absence of the thyroid hormones decreased the expression of CIC at transcriptional and post-transcriptional levels.

In conclusion, PUFA and starvation decrease the CIC gene promoter activity, whereas insulin and thyroid hormones enhance it. The regulation of the CIC transport activity is achieved not only by modulating the transcription of the CIC gene, but also by controlling mRNA stability and/or translation efficiency [70, 71, 73, 77]. Moreover, the composition of lipid domain surrounding the carrier protein is another factor capable of influencing the CIC transport activity.

## Mitochondrial CIC in pathophysiology

The multiple roles played by the mitochondrial CIC in cellular metabolism and physiology described in this review indicate that metabolism cannot be reduced to a series of processes that control the storage and use of energy. In this complex network, the molecule of citrate is involved not only in physiological, but also in pathological processes, such as inflammation and cancer, as well as in epigenetics [20, 24, 90].

In fact, it has been proposed that during inflammation, mitochondria of human leukocytes are addressed not only to produce ATP, but also to supply metabolic substrates, such as citrate, necessary for the synthesis of inflammatory molecules [91, 92]. The increase in the export of citrate from mitochondria leads to the production of acetyl-CoA and oxaloacetate. Acetyl-CoA is used to produce pro-inflammatory prostaglandin E2 (PGE2), whereas oxaloacetate (through cytosolic malate dehydrogenase and malic enzyme) produces NADPH needed for NO and ROS production. Moreover, cytosolic citrate also provides precursors for the synthesis of itaconate (an α,β-unsaturated dicarboxylic acid), an important mammalian metabolite which mediates the crosstalk between infection, immunity and metabolism.

The reprogramming of cellular metabolism is a hallmark of cancer cells, which rewire their metabolism to meet their demands for cancer development, progression and invasiveness. A different utilization of citrate has been observed in cancer cells, where citrate is predominantly exported outside mitochondria via the CIC to support lipid and macromolecules biosynthesis, as well as acetyl-CoA production [20, 90]. In this dynamic framework, the mitochondrial CIC may play an important role in the reprogramming energy metabolism through two mechanisms. Under nutrient-sufficient conditions, the CIC supplies acetyl units for fatty acid synthesis to promote tumor growth. During metabolic stress, it increases OXPHOS to protect cancer cells from energy stress-induced cell apoptosis [93]. In fact, the entry of malate into the mitochondria in exchange for citrate might impact upon mitochondrial respiration, the stability of mitochondrial membrane potential, as well as on glycolysis and on lipogenesis [94]. Moreover, cytosolic acetyl-CoA can be used in the mevalonate pathway leading to prenylation, which is a process involved in malignant transformation invasion and metastasis [95].

Acetyl-CoA is also the substrate for histone acetylation [20, 24] and is responsible for epigenetic consequences of metabolic reprogramming in cancer. This means that metabolic rewiring and epigenetic remodeling are closely linked and reciprocally regulate each other [96, 97]. By supplying acetyl units, the mitochondrial CIC could also play a pivotal role in genome maintenance as well as in adjusting the expression of several key regulatory enzymes. Therefore, a better understanding of the link among the mitochondrial CIC transport activity, cytosolic citrate levels, chromatin epigenetic modifications and genome stability will help to identify the molecular basis of human diseases. This will also be helpful in the investigation of new therapeutic approaches.

On the other hand, since 2013 until now, 24 mutations in SLC25A1 gene have been found, suggesting that CIC deficiency contributes to the severe neurodevelopmental symptoms of combined d-2- and l-2-hydroxyglutaric aciduria [98].

## Conclusions

It appears evident that the molecule of citrate has a dual role because it can act as a supplier of carbon source, in the form of acetyl units, for anabolic processes, and as a regulator, in the form of allosteric modulator, of several metabolic reactions. However, this charged molecule is unable to cross by itself the inner mitochondrial membrane which delimitates the site of its synthesis (mitochondrial matrix) from the site of its main utilization (cytosol). The mitochondrial CIC therefore catalyzes the flux of the molecule of citrate across the inner membrane and in doing so connects the glucose and amino acid catabolism to the fatty acid synthesis. The mitochondrial CIC has therefore a fundamental role inside the cells because it represents the molecular link between catabolic and anabolic reactions. In addition, the CIC activity is finely regulated in distinct nutritional and hormonal conditions and this finding underlines its role as a sensor of metabolic changes occurring inside the organism. In other words, it is becoming progressively clearer that the mitochondrial CIC does not play just the role of transporter of charged molecules across the lipid bilayer, but it is fully involved in the complex network of intermediary metabolism. Furthermore, the concentration of cytosolic citrate also depends on the activity of the transporter of this tricarboxylic acid located in the plasma membrane. Therefore, several molecules and proteins belonging not only to the same, but also to different metabolic pathways, need to be regulated in a fine and coordinated manner. It is also surprising the multiplicity of physiological processes in which the CIC is actively involved. These range from insulin secretion to sperm cells functioning and to DNA stability. It is also possible that the CIC is involved in further physiological processes not yet identified, whereas its involvement in inflammation and in some pathologies has been well established. We believe that it is important to integrate all these findings into a holistic view in order to identify the rationale behind this intriguing and complex framework. Hopefully, this will also help in understanding the molecular basis of the human pathologies in which the CIC is involved.

## Data Availability

Since it is a review article, the availability of data and material is not applicable.
